# Editorial: Endoscopic transorbital surgery for skull base tumors

**DOI:** 10.3389/fonc.2022.1042655

**Published:** 2022-10-27

**Authors:** Doo-Sik Kong, Kris. S. Moe

**Affiliations:** ^1^ Samsung Medical Center, Sungkyunkwan University, Seoul, South Korea; ^2^ Departments of Otolaryngology-Head & Neck Surgery and Neurological Surgery, University of Washington School of Medicine, Seattle, WA, United States

**Keywords:** transorbital, endoscopic, brain, skull base, meningioma, schwannoma, petrous apex, orbit

It is our pleasure to curate a group of 5 articles on transorbital neuroendoscopic surgery for skull base and brain tumors for presentation in this edition of *Frontiers in Oncology*. We have included some of the world’s foremost experts and research groups on the topic.

Transorbital neuroendoscopy is a relatively new field of surgery, with the first reports developing less than 15 years ago. The late development of this field is perhaps attributable to its anatomic location along the traditional borders of several specialties: otolaryngology – head & neck surgery, neurological surgery, and ophthalmology. Historically, these surgical specialties have confined their surgical domains within their anatomic regions of involvement: ophthalmologists within the orbit, otolaryngologists medial and inferior to the orbit, and neurological surgeons above the orbit. Introduction of technologies such as the endoscope and navigation guidance (analogous to GPS for instruments) however, has facilitated operating through traditional boundaries. And the expansion of other techniques such as transnasal and supraorbital endoscopic surgery, has provided predicate for multidisciplinary cooperation for advances in novel approaches.

A major attraction of the transorbital endoscopic approaches are their minimally disruptive nature. Rather than using long scalp incisions and muscle displacement, the transorbital approaches use small eye lid incisions developed for aesthetic oculoplastic surgery that are nearly invisible once healed. The pathways through the orbit are subperiosteal, requiring minimal retraction of orbital contents - 2.75 cm^3^ to reach the lateral cavernous sinus (Bly R, Ramakrishnan R, Ferreira M, and Moe K. Lateral Transorbital Neuroendoscopic Approach to the Lateral Cavernous Sinus. J Neurol Surg B Skull Base. 2014 Feb; 75(1): 11–17.) See **Figure 1**. Minimal volume of bone removal is needed to allow the passage of instruments to and from the surgical target, and retraction of the brain is significantly reduced from that of open craniotomy techniques. This results in minimally disruptive surgery with less patient discomfort, minimized hospital stay, and rapid return to the patient’s premorbid quality of life.

**Figure 1:**
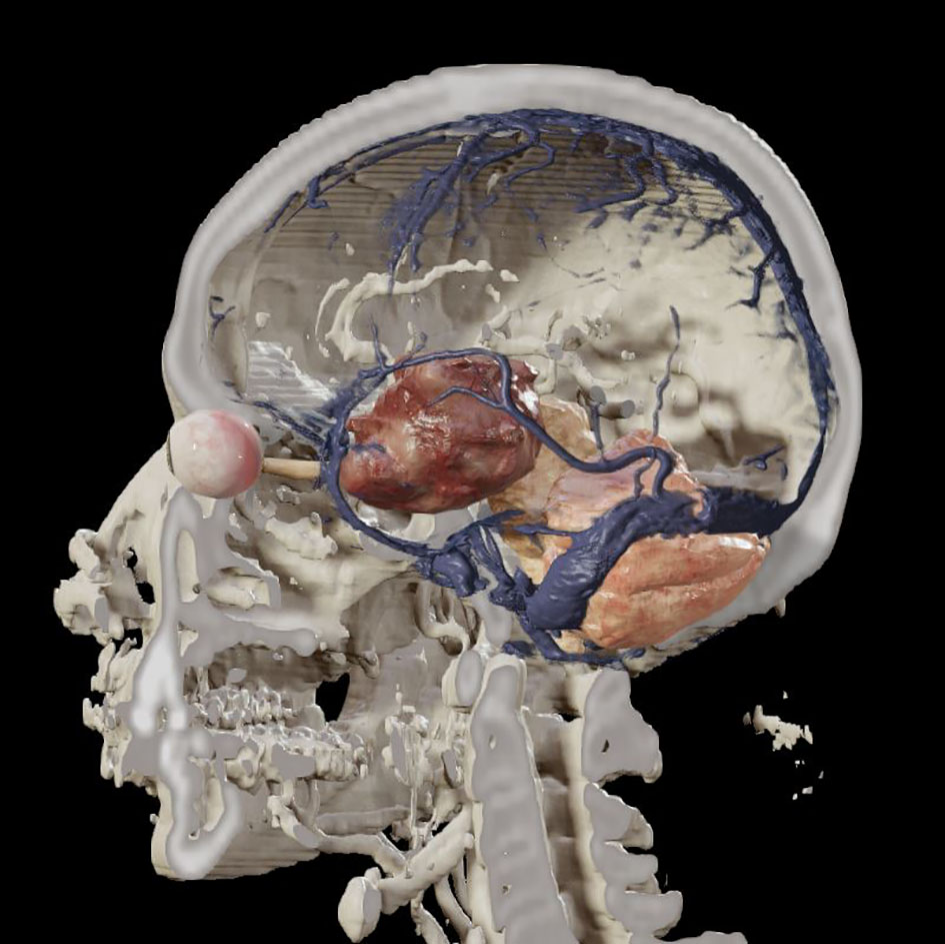
Coronal 3-D image depicting a tumor involving the left cavernous sinus that may be amenable to resection through a lateral transorbital endoscopic approach.

Endoscopic transorbital approaches have been described to address lesions in multiple locations including the orbit, the paranasal sinuses, the infratemporal fossa, the anterior and middle cranial fossae, and the posterior cranial fossa through the petrous apex. The approaches may be used as single pathways, or in combination with endoscopic transnasal or endoscopic/microscopic transcranial approaches in multiportal technique to optimize the ability to visualize and manipulate the surgical target.

For an innovative approach to become widely accepted as a standard treatment modality, it requires challenging a surgeon’s expertise and overcoming the resistance of experts familiar with the conventional approaches. The remarkable growth in number of publications, and more recently training courses, devoted to the field of transorbital endoscopic surgery attests to significant progress in this regard. This growth in information has contributed significantly to providing successful surgical outcomes by outlining appropriate surgical indications, development of a comprehensive body of anatomic knowledge on orbital and skull base anatomy (such as the meningo-orbital band, sagittal crest and mid-subtemporal ridge), and creation of curriculums for hands-on cadaver and simulation study to prepare the surgeon for embarking on these procedures.

In this edition of Frontiers in Oncology, there are contributions from experts in South Korea, Spain, Italy, the Netherlands, and Greece. Dallan et al. present an article titled “*Endoscopic-assisted transorbital surgery: Where do we stand on the scott’s parabola? personal considerations after a 10-year experience*”. Here they draw on their experience with endoscopic transorbital surgery to discuss the indications, pros and cons of the techniques, along with the requirements for attainment of surgical proficiency. They include a comparison of these techniques with classic transcranial approaches, as well as a discussion of possible future directions for the specialty.

Guizzardi et al. describe their conceptual analysis of the relevant anatomy in their article “*Endoscopic transorbital avenue to the skull base: Four-step conceptual analysis of the anatomic journey*”. They describe 4 steps in their developments: the study of bone landmarks on the dry skull, followed by cadaveric study; 3-dimensional CT pathway assessment; and assimilation of information gained by these analyses for detailed surgical planning.

Jung et al., present a cadaveric and clinical study of an approach to the cavernous sinus titled “*Endoscopic transorbital approach to the cavernous sinus: Cadaveric anatomy study and clinical application*”. In this manuscript they describe approaches to critical neurovascular structures through anatomic triangles. They compare endoscopic transnasal, endoscopic transorbital, and microscopic transcranial approaches and the applicability of each pathway based on target location within the CS and demonstrate the routes of the cranial nerves through each perspective

Lee et al. describe their work on the development and training outcomes of a novel 3-dimensional printed training model for endoscopic transorbital and transnasal surgery in their contribution titled “*Development of 3-dimensional printed simulation surgical training models for endoscopic endonasal and transorbital surgery*”. They include a validation analysis of the system including evaluation of the clinical value in a task-based hands-on dissection program, and note the importance of surgical simulation training due to the steep learning curve of these procedures.

“*Endoscopic transorbital extradural anterior clinoidectomy: A stepwise surgical technique and case series study*”, by Lim et al., describes their cadaver study of the complex anatomy of the anterior clinoid process and adjacent structures. Based on their analysis, they described 3 supporting roots of the structure, and developed 7 surgical steps for transorbital anterior clinoidectomy. They then performed a clinical study of the efficacy, safety and outcomes of the approach.

It is our hope that this collection of fascinating articles will stimulate further multidisciplinary interest and investigation in the exciting new field of transorbital neuroendoscopic surgery in the quest to provide highly effective, minimally disruptive surgical solutions to pathology in this challenging area.

## Author contributions

Both authors contributed to the writing and editing of this manuscript. D-SK contributed the figure. All authors contributed to the article and approved the submitted version.

## Conflict of interest

The authors declare that the research was conducted in the absence of any commercial or financial relationships that could be construed as a potential conflict of interest.

## Publisher’s note

All claims expressed in this article are solely those of the authors and do not necessarily represent those of their affiliated organizations, or those of the publisher, the editors and the reviewers. Any product that may be evaluated in this article, or claim that may be made by its manufacturer, is not guaranteed or endorsed by the publisher.

